# The Role of Spastin in Axon Biology

**DOI:** 10.3389/fcell.2022.934522

**Published:** 2022-07-05

**Authors:** Ana Catarina Costa, Monica Mendes Sousa

**Affiliations:** ^1^ Nerve Regeneration Group, Instituto de Biologia Molecular e Celular (IBMC), Instituto de Investigação e Inovação Em Saúde (i3S), University of Porto, Porto, Portugal; ^2^ Graduate Program in Molecular and Cell Biology, Instituto de Ciências Biomédicas Abel Salazar (ICBAS), University of Porto, Porto, Portugal

**Keywords:** axon growth, axon regeneration, axonal cytoskeleton, axonal transport, microtubules, microtubule severing enzyme, spastin

## Abstract

Neurons are highly polarized cells with elaborate shapes that allow them to perform their function. In neurons, microtubule organization—length, density, and dynamics—are essential for the establishment of polarity, growth, and transport. A mounting body of evidence shows that modulation of the microtubule cytoskeleton by microtubule-associated proteins fine tunes key aspects of neuronal cell biology. In this respect, microtubule severing enzymes—spastin, katanin and fidgetin—a group of microtubule-associated proteins that bind to and generate internal breaks in the microtubule lattice, are emerging as key modulators of the microtubule cytoskeleton in different model systems. In this review, we provide an integrative view on the latest research demonstrating the key role of spastin in neurons, specifically in the context of axonal cell biology. We focus on the function of spastin in the regulation of microtubule organization, and axonal transport, that underlie its importance in the intricate control of axon growth, branching and regeneration.

## Introduction

Neurons are highly specialized cells that form the building blocks of the nervous system. Owing to the various functions they carry, neurons present different morphologies, being highly polarized and compartmentalized (Dotti et al., 1988; Banker, 2018). The axon corresponds to the longest cell compartment in neurons, resembling a cable-like structure that ensures a reciprocate conduction of information from the cell body to the nerve terminal. In humans, neuronal cell bodies with an approximate size of 100 μm can give rise to up-to two-meter-long axons (Prokop, 2020; Prokop, 2021). In the course of a lifetime, axons cannot be replaced, with mammals losing up to 40% of their initial axon mass (Marner et al., 2003; Adalbert and Coleman, 2013; Calkins, 2013). Hence, axons are a key neuronal compartment in injury-induced trauma and neurodegeneration, and their decay with aging is on the basis of many neurodegenerative diseases (Medana and Esiri, 2003; Gaetz, 2004; Adalbert and Coleman, 2013; Salvadores et al., 2017). To prevent their loss and potentiate their ability to regenerate, it is fundamental to better understand the biology of axons.

Axonal microtubules (MTs) are the main tracks of axonal transport, allowing a continuous supplementation of the axon shaft with materials required to maintain its structure and function (Maday et al., 2014; Roy, 2014; Black, 2016; Guedes-Dias and Holzbaur, 2019; Roy, 2020). MTs are essential during neuronal development, and participate in the assembly of growth cones and elongation of axons until these meet their final targets (Kahn and Baas, 2016; Rodemer et al., 2020). The properties of axonal MTs are modulated by microtubule-associated proteins (MAPs) that control their dynamic instability, density, bundle formation, spacing and attachment to other cellular components (Akhmanova and Steinmetz, 2015; Bodakuntla et al., 2019). The severing enzymes spastin, katanin and fidgetin are MAPs that break MTs along their length leading to the formation of smaller MT fragments (Mcnally and Roll-Mecak, 2018; Kuo and Howard, 2021). Of note, spastin-deficient models show impaired axonal transport, supporting the important role of this MAP in modulating the transport of different organelles (Tarrade et al., 2006; Kasher et al., 2009; Fuerst et al., 2011; Rao et al., 2016; Wali et al., 2016; Plaud et al., 2017; Lopes et al., 2020; Wali et al., 2020). Furthermore, the administration of tubulin-binding drugs that interfere with MT stability restore axonal transport in spastin-deficient models (Denton et al., 2014; Fan et al., 2014; Wali et al., 2016; Wali et al., 2020). In addition to their well-known importance during neuronal development (Ahmad et al., 1999; Kapitein and Hoogenraad, 2015; Baas et al., 2016), MT severing enzymes also play a fundamental role in nervous system physiology and in the course of axon regeneration (Qiang et al., 2006; Wood et al., 2006; Stone et al., 2012; Yang et al., 2013; Chen et al., 2014; Rao et al., 2016; Austin et al., 2017; Matamoros et al., 2019).

In this review, we discuss the above findings focusing on the role of spastin in the regulation of MT dynamics, and control of axonal transport. We start by detailing MT organization and regulation in axons, focusing on the role of severing enzymes. We then explore the biology of the severing enzyme spastin. We finish by discussing how spastin underlies important axonal events, such as synapse maintenance, MT repair, axonal transport, branching and (re)growth.

### Microtubule Organization and Dynamic Instability

MTs are one of the major components of the cell cytoskeleton where they play vital roles in setting cell shape, motility, mitosis, and intracellular transport. They are composed of α- and β-tubulin heterodimers that assemble in a circular shape, of approximately 24nm, producing a hollow tube of usually 13 protofilaments (Akhmanova and Steinmetz, 2008). The organization of α- and β-tubulin in a head to tail arrangement makes MTs polar structures, which confers directionality through their length (Gudimchuk and Mcintosh, 2021). The polarity of MTs also results in different kinetics of subunit addition and loss at the 2 MT ends, being the faster growing end known as the MT “plus-end” and the slower growing end known as the MT “minus-end” (Akhmanova and Steinmetz, 2019). Motor proteins, kinesin and dynein, read this polarity in order to drive cell transport. Conventional kinesins, with their motor domain located at the amino terminal, move towards the MT plus-end while dynein moves towards the MT minus-end (Tas et al., 2017; Kelliher et al., 2019). In vertebrate neurons, axons have most plus-end MTs facing the axon terminal, while dendrites contain a combination of plus-end-out and minus-end-out MTs, whose proportions change between organisms and throughout neuronal development (Luders, 2021).

Most cellular MTs exhibit cycles of growth, shrinkage (catastrophe) and regrowth (rescue), which are overall termed as MT “dynamic instability” (Desai and Mitchison, 1997; Mitchison and Kirschner, 1984). This important MT feature allows these dynamic structures to explore the intercellular space, binding and cross-talking with other intracellular elements (Pacheco and Gallo, 2016; Dent, 2017), and the extracellular space, participating in cell migration, growth, and regeneration. The MT dynamic instability is better explained by the “GTP cap model” proposed by Mitchison and Kirschner (Mitchison and Kirschner, 1984). This process starts when free tubulin binds to guanosine triphosphate (GTP). Shortly after incorporation into the MT lattice, GTP-bound β-tubulin is hydrolyzed into guanosine diphosphate (GDP), with the majority of the growing MT being composed of GDP-bound tubulin with only a small region at the plus-tip containing newly incorporated GTP-bound tubulins (Mitchison and Kirschner, 1984; Akhmanova and Steinmetz, 2015). Because GDP-bound tubulin is unstable and easily disassembles, MT persistent growth and stability relies on the GTP-bound tubulin cap. As soon as MTs lose this GTP-bound tubulin cap, GDP-bound tubulin is exposed, and the MT undergoes catastrophe. Despite occurring at both MT ends, this phenomenon is mainly prevalent at the MT plus-end, where different MAPs (collectively called plus-tip proteins) associate to regulate the dynamic instability of this tip, tunning the MT in accordance with different cellular needs. Contrarily to MT plus-end, MT minus-ends are less dynamic, being capped by a third tubulin isoform, γ-tubulin, which functions as a MT nucleator and reduces dynamic instability by blocking the exchange of tubulin dimers (Wiese and Zheng, 2000; Kollman et al., 2011). Whilst the MT plus-ends explore the cellular space and interact with intracellular structures, the MT minus-ends determine the geometry of the MT network (Akhmanova and Steinmetz, 2015; Martin and Akhmanova, 2018). MT nucleation is dependent on the presence of microtubule-organizing centers (MTOCs), which bring together and position tubulin, usually mediated by means of centrosomes (Akhmanova and Kapitein, 2022). While neuronal progenitors display centrosomes, throughout development neurons undergo centrosome inactivation, displaying a non-centrosomal organisation in adulthood (Stiess et al., 2010; Sulimenko et al., 2017). How acentrosomal MTs are generated in neurons is still poorly understood. Nonetheless, an important acentrosomal mechanism of MT nucleation is MT branching, where new MTs are generated from the lattice of a pre-existing MT. This nucleation mechanism is important for the formation of axonal and dendritic MTs in both mammal and *drosophila* neurons ( Yalgin et al., 2015; Sanchez-Huertas et al., 2016; Cunha-Ferreira et al., 2018 ).

One of the major roles of MTs is to sustain intracellular transport, being fundamental in neurons, where axonal processes can extend up-to two-meter-length in large mammals. This requires a close regulation of the MT cytoskeleton and molecular motors to maintain important neuronal processes such as axon growth or regeneration, synaptogenesis, synaptic transmission, and plasticity (Guillaud et al., 2020). Intracellular transport is an ATP-dependent mechanism that shuttles diverse cargoes in a bidirectional way. In axons, kinesins drive anterograde transport, delivering synaptic vesicles, mitochondria, lysosomes, cytoplasmic proteins and mRNAs from the cell body towards growth cones and synapses (Terenzio et al., 2017). On the opposite direction, cytoplasmic dynein drives the retrograde transport of signaling endosomes, autophagosomes, and injury signals (Rishal and Fainzilber, 2014; Reck-Peterson et al., 2018; Olenick and Holzbaur, 2019). In mammals, there are 45 genes that encode for different kinesins, but only 38 are expressed in the nervous system, being classified in 15 different families (Hirokawa and Tanaka, 2015). Members of the kinesin-1, -2, -3, and -4 families mediate transport in neurons while the others participate in cellular processes such as mitosis and cytoskeletal remodeling (Hirokawa et al., 2009; Silverman et al., 2010). In contrast, retrograde axonal transport is accomplished by a single form of cytoplasmic dynein that is a large multi-subunit motor complex (Reck-Peterson et al., 2018).

Considering the importance of MTs to drive axonal transport, their length and density have a direct impact in the overall success of this process. Several studies indicate that axonal transport defects occur not only in a multitude of neurodegenerative diseases but may also underlie the failure of axon regeneration (Petrova and Eva, 2018; Guillaud et al., 2020; Guo et al., 2020). MTs cover the length of an axon in an overlapping manner (Chalfie and Thomson, 1979) and organelles being transported along them need to pause at the polymer end to rebind to another MT (Yogev et al., 2016). In this regard, in *C. elegans*, increased MT density is linked to increased axonal transport, since cargo pause time is inversely correlated with the abundance of MTs (Yogev et al., 2016). Furthermore, cargo run length is set by MT length. Supporting this data, a study using a 3D Monte Carlo simulation to model vesicular transport in the axon shows that closely spaced parallel MTs enable cargoes to simultaneously engage motors on more than one MT, therefore enhancing motor activity and increasing axonal transport (Wortman et al., 2014).

Recently, the idea that MT dynamics is a phenomenon exclusive of the MT tips was challenged. Different *in vitro* experiments demonstrated that lattice defects occur along the MT, which originate either spontaneously (Schaedel et al., 2019) or due to mechanical stress (Schaedel et al., 2015), motor movement (Triclin et al., 2021), or enzymatic activity (Vemu et al., 2018). Importantly, MTs have a self-repair mechanism that allows them to restore these damaged sites by incorporation of free tubulin, presumably in a GTP-bound state (Schaedel et al., 2015; Aumeier et al., 2016; Vemu et al., 2018; Schaedel et al., 2019). Aside from *in vitro* experiments, lattice defects were also observed in cell free extracts of *Xenopus* eggs (Chretien et al., 1992) and in mouse embryonic hippocampal neurons (Atherton et al., 2018). Different MAPs, such as molecular motors and severing enzymes, can induce damage to the MT lattice. The movement of molecular motors along MTs can provoke lattice damage by pulling out tubulin heterodimers (Vandelinder et al., 2016; Triclin et al., 2021). A recent *in vitro* study employing a two-color gliding assay shows that both kinesin-1 and dynein produce damage on MTs that is then repaired by free tubulin incorporation at these sites (Triclin et al., 2021). Interestingly, by varying the duration of the gliding step, it was found that while the size of the repairs remained constant, their frequency along the shaft increased, implying that molecular motors are continuously generating new sites of damage that are then self-repaired. Nonetheless, despite the mechanisms by which molecular motors breakdown the MT lattice are still unknown, this raises the possibility that these motors trigger the selective stabilization of the MTs they are walking on, defining preferential tracks in MT networks (Thery and Blanchoin, 2021). Severing enzymes change the conformation of the MT lattice by destabilizing and removing tubulin heterodimers (Mcnally and Roll-Mecak, 2018; Kuo and Howard, 2021). By combining single-molecule total internal reflection fluorescence microscopy and electron microscopy, it was observed that similarly to molecular motors, the action of severing enzymes generates nanoscale damage throughout the MT lattice that is counteracted by spontaneous incorporation of GTP-bound tubulin, forming GTP-islands (Vemu et al., 2018). Newly incorporated GTP-islands also create points along MTs where these can be rescued from depolymerization and regrowth. Interestingly, MT damage inflicted by spastin can be recognized by the MAP Sjogren’s syndrome nuclear autoantigen-1 (SSNA1/NA14), which coats the MTs protecting them against spastin’s severing activity (Lawrence et al., 2021). MT repair is particularly prevalent at MT crossovers and bundles (Aumeier et al., 2016), where MT severing enzymes are also present in high levels (Lindeboom et al., 2013; Zhang et al., 2014). How impaired lattice repair contributes to the disease phenotypes in patients with spastin and katanin mutations is still unknown.

### Tubulin Isotypes and Post-Translational Modifications

MTs exhibit a complex range of behaviors and properties amongst distinct species, cells and even within cellular compartments. Such complexity is introduced by the “tubulin code”, a selective combination of different tubulin isotypes and tubulin post-translational modifications (PTMs) that regulate MT structure and dynamics, having a direct impact in its cellular function (Gadadhar et al., 2017).

A broad variety of tubulin isotypes are encoded by different tubulin genes, differing across species (Janke and Magiera, 2020). While mice express seven α- and β-tubulin genes, humans express eight α- and nine β-tubulin genes. Members within each tubulin sub-family typically show >90% homology (Guedes-Dias and Holzbaur, 2019), possessing a highly conserved core structure and an unstructured negatively charged C-terminal tail, which varies considerably (Nogales, 2001; Sirajuddin et al., 2014). The C-terminal tail, which decorates the MT exterior, can be further modified by different PTMs that will fine-tune their affinity towards different MAPs (Nogales, 2001), influencing several cellular mechanisms (Sirajuddin et al., 2014; Parker et al., 2022). Engineered α- and β-tubulin heterodimers with distinct C-terminal tails and PTMs are also known to regulate motor protein velocity and processivity (Sirajuddin et al., 2014). Initial *in vitro* studies showed that different tubulin isotypes regulate MT dynamics differently (Lu and Luduena, 1994; Panda et al., 1994), with recent work showing that different isotypes have distinct dynamic properties (Vemu et al., 2016; Vemu et al., 2017; Diao et al., 2021).

Despite the variety of tubulin isotypes, they freely blend to form MTs with different proportions of each isotype (Lewis et al., 1987). Of note, βIII-tubulin, encoded by the *TUBB3* gene, is present in neuronal MTs (Joshi and Cleveland, 1989; Lee et al., 1990), being a classic neuronal marker (Jiang and Oblinger, 1992). However, some tubulin isotypes are not interchangeable, being tissue and cell specific. As an example, *in vivo* studies in embryonic *Tubb3*-knockout mice show defects in neuronal migration that cannot be rescued by other tubulin isotypes (Saillour et al., 2014).

In addition to the existence of several tubulin isotypes, the “tubulin code” further includes distinct tubulin PTMs that can modify the core or the C-terminal tail of tubulin isotypes, including acetylation, detyrosination/tyrosination, phosphorylation, polyglutamylation, polyamination, (poly)glycylation, and, although less abundant, palmitoylation, ubiquitylation, sumoylation, nitrosylation, nitration and methylation. A fine regulation of these modifications controls MT mechanical properties and interaction with other MAPs (Janke and Magiera, 2020). Different subsets of MTs with different tubulin PTMs coexist in the same cell compartment, which raises many questions regarding how these different MT subsets are established and maintained (Katrukha et al., 2021). In this review we will mainly focus on tubulin acetylation, tyrosination/detyrosination and polyglutamylation given their fundamental role in regulating MT dynamics and axonal transport.

MTs acetylated at the residue Lys40 of α-tubulin were first identified in the flagella of *Chlamydomonas sp* (Lhernault and Rosenbaum, 1983). In neurons, tubulin acetylation at Lys40 is enriched in the axon initial segment and axon shaft and is a rare PTM in the growth cone and synapses (Cappelletti et al., 2021). In contrast to other PTMs, acetylated tubulin at Lys40 is located in the lumen of stable MTs, being regulated in a reversible way, by the acetyltransferase αTAT1 (Shida et al., 2010) and the deacetylating enzymes histone deacetylase 6 (HDAC6) and sirtuin 2 (SIRT2) (Hubbert et al., 2002; North et al., 2003). Acetylation directly impacts MT dynamics by interfering with MT stability as confirmed by studies in *C. elegans* where ablation of the acetyltransferase MEC-17 (the *C. elegans* orthologue of αTAT1) induces loss of α-tubulin acetylation in neurons and promotes microtubule instability in axons (Neumann and Hilliard, 2014). Despite its important role in modulating MT stability, a novel concept regarding MT acetylation is emerging. By weakening the lateral interactions between protofilaments, MT acetylation enhances MT flexibility, conferring resistance against repeated mechanical stresses and, as a result, increasing MT longevity (Portran et al., 2017; Xu et al., 2017).

Tubulin tyrosination was the first tubulin PTM identified after discovering that α-tubulin in rat brain homogenates could incorporate tyrosine in a translation-independent manner (Arce et al., 1975a; Arce et al., 1975b). Shortly after this discovery, it was found that tyrosinated tubulin can be detyrosinated (Hallak et al., 1977), a reversible cycle that occurs at the C-terminal tail of most α-tubulin isotypes. Unlike other PTMs that appear on proteins other than tubulins, tyrosination/detyrosination is specific of α-tubulin. While tubulin tyrosination is catalyzed by the tubulin tyrosine ligase, which acts preferentially on soluble tubulin (Kreis, 1987; Ersfeld et al., 1993), tubulin detyrosination is catalyzed by vasohibin and its interacting partner SVBP (Aillaud et al., 2017; Nieuwenhuis et al., 2017). Neurons are mainly decorated with detyrosinated tubulin in the axon shaft and tyrosinated tubulin in the distal regions of axons, contiguous with the growth cone (Brown et al., 1993; Song and Brady, 2015). Since detyrosinated tubulin is found in long-lived MTs, it is considered as a marker of MT stability (Peris et al., 2009; Sirajuddin et al., 2014), being resistant to disassembly by the MT depolymerizing drug nocodazole (Baas and Black, 1990). On the other hand, tyrosinated MTs are mainly present in the labile domain of MTs. Ultimately, tubulin tyrosination and detyrosination also regulate transport as the molecular motors kinesin-1 (KIF5A) and kinesin-2 (KIF17) display a preference for detyrosinated MTs (Dunn et al., 2008; Hammond et al., 2010). whereas kinesin-3 (KIF1A) and kinesin-5 (KIF11) prefer tyrosinated MTs (Kahn et al., 2015; Tas et al., 2017). Tyrosination also affects the movement of dynein, controlling the transport initiation by the dynein-dynactin complex via CLIP-170 in dorsal root ganglion neurons (Nirschl et al., 2016).

Tubulin glutamylation is the most abundant PTM in neurons and is characterized by the addition of glutamate to the γ-carboxy group of another glutamate on both α and β-tubulin (Edde et al., 1990; Rudiger et al., 1992). The addition of glutamates on tubulins consists of two steps: the initiation step, during which the first residue of glutamate is added on the carboxy-terminal domain of tubulin, and the elongation step that leads to the formation of a polyglutamyl side chain (Devambez et al., 2017). These PTMs are catalyzed by nine different tubulin-tyrosine ligase like enzymes (TTL1, -2, -4, -5, -6, -7, -9, -11, and -13), each one with preference for a different tubulin isotype and for the initiation or elongation of glutamate chains (Sferra et al., 2020). In the mammalian nervous system, two TTLLs are expressed in high abundance: TTLL1 and TTLL7. Being a reversible tubulin PTM, the deglutamylation is catalyzed by four different cytosolic carboxypeptidases (CCP 1, 4, 5, and 6), which confers a fine-tuning control over the MT cytoskeleton (Janke et al., 2005; Ikegami et al., 2006; Van Dijk et al., 2007; Kimura et al., 2010; Rogowski et al., 2010; Tort et al., 2014). Tubulin polyglutamylation modulates the binding of different kinesins and MAPs to MTs (Larcher et al., 1996). Finally, polyglutamylation also affects transport, as in mice loss of polyglutamylated α-tubulin in neurons is associated with decreased MT binding affinity of KIF1A (Ikegami et al., 2007). This dysregulation of polyglutamylation alters the proportion of synaptic vesicles being transported towards the CA1 region of the hippocampus, changing synaptic transmission (Ikegami et al., 2007). As detailed below, axonal transport is also sensitive to polyglutamylation because of spastin-mediated severing. Changes in the polyglutamylated surface of MTs modulate the rate of severing, leading to alterations in MT tracks (Ikegami et al., 2007; Lacroix et al., 2010; Valenstein and Roll-Mecak, 2016).

Taken together, all the above evidence supports that the complex pattern of the “tubulin code” regulates MT dynamic behavior, MT mass and ultimately, axonal transport.

### Microtubule Severing Enzymes in Neuronal Biology

An ever-growing amount of evidence supports the fundamental role of severases in axonal physiology and pathology. MT severing enzymes generate internal breaks in MTs. This action is mediated by three conserved enzymes of the AAA+ (adenosine triphosphatases associated with various cellular activities) family - katanin, fidgetin and spastin (Mcnally and Roll-Mecak, 2018). Severases, in contrast to other MT depolymerases, bind to and generate internal breaks in the MT lattice and not at the MT end (Howard and Hyman, 2007; Roll-Mecak and Mcnally, 2010). Originally, it was thought that MT severing was a destructive process leading to decreased MT mass (Mcnally et al., 2000; Errico et al., 2002; Yu et al., 2005). Severases disassemble MTs by exposing their GDP region, which may lead the newly created plus-end to depolymerize due to the absence of a GTP cap to prevent this fate, which would thereby lead to a decrease in MT mass. Paradoxically, as will be discussed below, several *in vivo* and *in vitro* studies demonstrated the opposite. When MT severing enzymes were disrupted, the absence of their activity did not result in an increase of MT mass but, unexpectedly, in a reduction (Sherwood et al., 2004; Srayko et al., 2006; Wood et al., 2006; Stewart et al., 2012). In the following paragraphs, the importance of severases, in particular that of spastin, in axon biology will be discussed.

### Katanin: A Key Microtubule Severing Enzyme During Developmental Axon Growth

Katanin was the first MT severing enzyme to be characterized. It is composed by two subunits, the catalytic subunit p60 (encoded by *KATNA1*) and the regulatory subunit p80 (encoded by *KATNB1*) (Mcnally and Roll-Mecak, 2018). Katanin mutations cause microcephaly, seizures, and severe developmental defects (Hu et al., 2014; Mishra-Gorur et al., 2014; Yigit et al., 2016). This severing enzyme recognises polyglutamylated and acetylated tubulin on stable MTs (Lacroix et al., 2010; Sudo and Baas, 2010; Shin et al., 2019). In mouse hippocampal neurons, katanin binds preferentially to acetylated MTs in dendrites (Sudo and Baas, 2010). However, it does not show the same preference for axonal MTs, possibly due to their higher content in tau, which protects MTs from katanin-mediated severing (Bradke and Dotti, 2000; Qiang et al., 2006).

The levels of katanin in the brain change throughout development, being high during axon growth and falling as soon as axons reach their target cells (Karabay et al., 2004). Microinjection of cultured sympathetic neurons with a polyclonal antibody against the p60 subunit of katanin blocks its severing activity and inhibits axon outgrowth (Ahmad et al., 1999). This suggests that katanin may be involved in MT regulation in developing neuronal processes (Ahmad et al., 1999). In support of this hypothesis, in hippocampal neurons, p60-katanin promotes neurite outgrowth and facilitates neurite branching (Chen et al., 2014). Ubiquitin-dependent proteolysis regulates katanin levels in neurons as well as its role in axon growth, via the enzymes ubiquitin carboxyl-terminal hydrolase 47 (USP47) and C-terminus of Hsp70-interacting protein (CHIP) (Yang et al., 2013). CHIP ubiquitinates katanin p60 for subsequent degradation by the 26S proteosome, leading to a decrease in its levels and a decline in hippocampal axon growth. Conversely, USP47 reverses the ubiquitination process by removing ubiquitin from katanin p60, resulting in its accumulation and increased hippocampal axon growth (Yang et al., 2013).

Overall, the above data strongly supports the role of katanin in the modulation of the MT cytoskeleton in developing axons, enabling their robust developmental growth.

### Fidgetin: A Target to Enhance Axon Growth and Regeneration

Fidgetin was initially found by positional cloning as the gene mutated in spontaneous mutant mice with a characteristic “head-shacking-and-circling” behavior (Cox et al., 2000). In contrast to katanin and spastin, vertebrate fidgetin targets the non-acetylated labile domain of MTs, where tyrosinated tubulin is highly present (Leo et al., 2015). Vertebrate fidgetin functions during development to tamp back the elongation of the labile domain of MTs so that they can assemble in a regulated fashion (Leo et al., 2015). Consequently, overexpressing fidgetin suppresses normal axon elongation whereas depletion of fidgetin results in longer labile MTs and longer axons (Leo et al., 2015). Accordingly, in *vitro* cultures of primary dorsal root ganglion neurons, downregulation of fidgetin fosters axon regeneration in both permissive and inhibitory environments (Austin et al., 2017). *In vivo,* in dorsal root ganglion neurons, knocking-down fidgetin results in enhanced axon regeneration across the dorsal root entry zone after a dorsal root crush (Matamoros et al., 2019). These studies establish fidgetin as a novel therapeutic target to enhance axon regeneration. Similarly, in rat astrocytes, fidgetin preferentially targets the dynamic, tyrosinated MTs playing an important role in the migration of these glial cells (Hu et al., 2017). Additionally, fidgetin-like 2, a member of the fidgetin family, is a negative regulator of axon regeneration, suppressing axon growth by selectively severing dynamic MTs in the distal axon shaft and growth cone of murine adult dorsal root ganglion neurons (Baker et al., 2021). Fidgetin-like 2 depleted neurons show high rations of tyrosinated tubulin and low rations of acetylated tubulin in the distal neurite regions. Therefore, downregulation of fidgetin and fidgetin-like 2 are emerging as new targets to enhance axon (re)growth.

### Spastin: From Structure and Function to the Regulation of MT Dynamics, Axonal Transport and Axon Growth

Spastin was originally found in the context of hereditary spastic paraplegia (HSP). Similarly, to katanin, spastin also demonstrates preference for stable MTs, with its catalytic activity being dependent on polyglutamylated tubulin (Lacroix et al., 2010; Valenstein and Roll-Mecak, 2016; Han et al., 2020). Spastin displays a graded response to the size of the tubulin glutamate-side chain, enhancing its catalytic activity when the side chain increases up to eight glutamate residues and progressively decreasing catalytic activity as this number increases (Valenstein and Roll-Mecak, 2016). As detailed below, spastin’s severing activity is dependent on its oligomerization in a hexamer.

Spastin has four protein isoforms. The two major isoforms, spastin M1 and spastin M87, occur as a consequence of alternative start sites. The full-length spastin M1 isoform (68kD) has 616 amino acids while the shorter isoform, spastin M87 (60kD), lacks the first 86 amino acids (Claudiani et al., 2005). Despite being simultaneously synthesized, the first AUG is surrounded by a weak Kozak sequence, which results in a preferred translation of the second AUG (Claudiani et al., 2005; Mancuso and Rugarli, 2008). Furthermore, spastin M1 and M87 can undergo alternative splice at exon four leading to the formation of two additional isoforms, M1∆4 (64kD) and M87∆4 (55kD) (Salinas et al., 2007; Fink, 2013). Nevertheless, these two isoforms are the least abundant with no specific functions being reported so far.

Full-length spastin (spastin M1) is composed of four functional domains: the hydrophobic domain (amino acids 1–87), with high affinity for membranes; the MT-interacting and trafficking (MIT) domain (amino acids 116–194) that is essential for binding to endosomal sorting complexes required to transport ESCRT-III proteins that participate in endosomal trafficking; the MT binding domain (MTBD) (amino acids 270–328), which mediates the interaction between spastin and tubulin, underlying its severing activity; and the adenosine triphosphatases associated with diverse cellular activities (AAA) domain (amino acids 342–599), necessary for the assembly of spastin in an hexamer and for the severing of MTs (Connell et al., 2009; Qiang et al., 2019a). Together, the MTBD and the AAA domain are indispensable for spastin MT severing in an ATP hydrolysis-dependent manner (Mcnally and Roll-Mecak, 2018).

The 86 amino acids that compose spastin M1 form an N-terminal hydrophobic region that spans the outer leaflet of endoplasmic reticulum (ER) membranes and lipid droplets, regulating the activity of these organelles (Arribat et al., 2020). As spastin M87 lacks this region, it is more soluble than spastin M1 (Claudiani et al., 2005). The N-terminal region of spastin M1 plays an important function in ER shaping (Park et al., 2010), lipid droplet metabolism (Chang et al., 2019; Arribat et al., 2020), membrane remodelling (Guo and Xu, 2015; Vietri et al., 2020), endosomal fission (Kasher et al., 2009; Allison et al., 2013; Allison et al., 2017; Leo et al., 2017; Connell et al., 2020) and fast axonal transport (Solowska et al., 2008; Kasher et al., 2009; Leo et al., 2017). The function of spastin M87 is less well explored. However, some studies support an important role in axonal transport (Connell et al., 2020) and its recruitment to endosomes in a MIT domain-dependent manner (Renvoise et al., 2010; Connell et al., 2020). Both spastin isoforms have the capacity to sever MTs and to hetero-oligomerize (Roll-Mecak and Vale, 2008). Two studies propose that efficient hexamer formation involves the nucleation of spastin M1 at the ER-endosome contact, followed by rapid recruitment of spastin-M87 from the cytosol (Allison et al., 2017; Connell et al., 2020).

With reference to their location, spastin M87 is more abundant and ubiquitously expressed (Claudiani et al., 2005; Wood et al., 2006), whereas spastin M1 is enriched in the adult brain and spinal cord (Claudiani et al., 2005; Solowska et al., 2008). At the cellular level, spastin M87 is present both in the cytoplasm and the nucleus, while spastin M1 is exclusively present in the cytoplasm (Claudiani et al., 2005). This distribution is specified by two nuclear localization signals (NLS) and two strong overlapping nuclear export signals (NES), located between amino acids 50–87, that are unique to spastin M1 (Claudiani et al., 2005). A recent study shows that both isoforms have an additional NES sequence, located between amino acids 195–204 that spans the MIT domain and the exon four region (Sakoe et al., 2021). Moreover, mutants for this newly identified NES reduce spastin M87 cytoplasmic localization and MT fragmentation, whereas the same mutation on spastin M1 had only minor effects on its localization and MT severing. This shows that the NES sequence that spans amino acids 195–204 is fundamental for spastin M87 cytoplasmic localization and MT severing.

### Spastin Severing Model and Nucleation-Like Activity

Spastin exists as a monomer at physiological concentrations, even when bound to ATP (Roll-Mecak and Vale, 2008). Its severing activity is dependent on the oligomerization of spastin in a hexamer through the AAA domain. This hexamerization is stimulated in the vicinity of MTs, ensuring that it is only activated close to its substrate (Roll-Mecak and Vale, 2008; Eckert et al., 2012; Wen and Wang, 2013; Zehr et al., 2017). The current hypothesis for tubulin extraction by spastin and other severases proposes that the ring formed within the hexamer is positively charged and engages with the negatively charged C-terminal tail of tubulin that decorates the MT surface. Spastin binds to glutamylated tubulin at the C-terminal tail of α- and β-tubulin in a biphasic way, which enables a substrate-regulated and spatially controlled severing (Lacroix et al., 2010; Valenstein and Roll-Mecak, 2016). This interaction mechanically extracts tubulin from the MT lattice using ATP, which repeatedly generates internal breaks that can lead to the complete rupture of this cytoskeleton structure (White et al., 2007; Roll-Mecak and Vale, 2008; Sandate et al., 2019; Han et al., 2020).

How severing enzymes accomplish MT nucleation is still unknown. Yet, two major models have been put forward (Vemu et al., 2018; Kuo et al., 2019). Vemu and colleagues suggest that spastin and katanin actively extract tubulin dimers out of the MT, creating nanodamage along the MT lattice. This action is neutralized by spontaneous *de novo* incorporation of GTP-tubulin dimers from the soluble pool. Depending on the local balance between tubulin extraction and passive repair, MTs can either be rejuvenated with GTP-tubulin islands that stabilize them against depolymerization, or the severing is completed and the newly severed MT emerges with a high density of GTP-tubulin that prevents its depolymerization, amplifying MT mass (Vemu et al., 2018). However, it is not clear whether this new incorporated tubulin at the GTP-islands is not hydrolyzed to GDP-tubulin thereby failing to prevent the depolymerization of MTs. Nevertheless, this hypothesis raises the possibility that severing enzymes might also serve a function of quality control proteins in hyperstable MTs. Spastin can accumulate at the plus-tip of shrinking MTs slowing shrinkage and promoting rescue through unknown multivalent interactions with shortening MT tips (Kuo et al., 2019). The authors speculate that these interactions can slow down the shrinkage of GDP-tubulin, allowing sufficient time for the formation of a new GTP-cap in the newly formed MT plus-tip. Additional mechanistic studies are necessary to test these two models. Currently, it is not clear why severases lead to MT regrowth in some situations and to MT disassembly in others. One possible explanation is the interaction of these enzymes with other MAPs (Kapitein and Hoogenraad, 2015; Baas et al., 2016). In fact, MT assembly is promoted by rescue factors such as CLIP-associating proteins (CLASPs) or MT stabilizers such as tau (Yu et al., 2008; Lindeboom et al., 2019).

### Regulation of Spastin Levels and Activity

A delicate balance of spastin levels and activity is necessary for its appropriate biological function (Riano et al., 2009; Stone et al., 2012; Havlicek et al., 2014). Spastin’s expression, as well as its stability and activity, are spatiotemporally regulated, not only at the transcriptional level, but also through PTMs and through the control of MAPs. In neuronal cells the expression of spastin is positively regulated by the transcription factors NRF1 and Sox11, whereas the neuronal transcription factor Elk1 decreases both katanin p60 and spastin expression (Kelle et al., 2019). Spastin is also negatively regulated by various microRNAs in neuronal cells (Henson et al., 2012; Jiang et al., 2020), being inhibited by miR-30, which results in hippocampal neurons with reduced MT severing and neurite outgrowth (Jiang et al., 2020). Other miRNAs negatively regulate the expression of spastin in neuronal cells, namely miR-182 and miR-96 (Henson et al., 2012). miR-33 is also a specific spastin negative regulator in human cells. Interestingly, inhibition of this miRNA leads to improved HSP disease phenotypes in cortical neurons, specifically in what concerns neurite length and branching (Nakazeki et al., 2019).

The activity of spastin can also be modulated at the post-transcriptional level. Homeodomain-interacting protein kinase 2 (HIPK2), a highly conserved multifunctional tyrosine-regulated serine/threonine kinase expressed in the nervous system, was the first spastin kinase identified (Pisciottani et al., 2019; Sardina et al., 2020). The phosphorylation of spastin at Ser268 inhibits spastin polyubiquitination and prevents its neddylation-dependent proteasomal degradation (Sardina et al., 2020). Spastin levels are thus controlled by a fine-tuning balance between Ser268 phosphorylation and polyubiquitination/degradation processes. A more recent study identified Ser210 as another site where spastin can be phosphorylated (Li et al., 2021), decreasing its binding to MTs and its severing activity. Spastin is also SUMOylated at Lys427, which alters its severing activity, promoting MT stability and dendritic spine development in hippocampal neurons (Ji et al., 2020).

In addition to post-translational modifications, regulation of spastin by MAPs, as is the case of Tau, SSNA1/NA14 and collapsin response mediator proteins (CRMPs), can also occur. Tau increases the rate of MT polymerization, inhibiting depolymerization and strongly suppressing catastrophe (Drechsel et al., 1992; Prezel et al., 2018). In axons, Tau forms oligomers on the outer MT surface (Al-Bassam et al., 2002). Tau can induce MT stability by regulating MT interaction with other MAPs, namely katanin and spastin (Qiang et al., 2006; Siahaan et al., 2019; Tan et al., 2019). Overexpression of tau protects MTs against the severing action of katanin and spastin (Qiang et al., 2006; Yu et al., 2008), while loss of tau in neurodegenerative diseases, such as Alzheimer’s, indirectly leads to increased spastin activity (Zempel and Mandelkow, 2015). Recently, two studies have shown that tau nucleates and expands in denser regions above MTs, creating small regions along the MT termed “tau condensates” or “tau islands” (Siahaan et al., 2019; Tan et al., 2019). Interestingly, these studies show that spastin and katanin are largely excluded from these tau islands, which protect the underlying MT from severing. It was further demonstrated that MT regions not protected by tau islands are destroyed by severing activity. In this respect, tau islands act as selective permissive barriers for severing enzymes.

SSNA1/NA14 is a MAP with important functions in developing neurons, where it promotes axon elongation and branching (Goyal et al., 2014; Basnet et al., 2018). It self-assembles in higher-order fibrils that bind longitudinally along stabilized MTs, inducing their branching and nucleation (Basnet et al., 2018). Using *in vitro* reconstitution techniques with purified protein components and TIRF microscopy, progressive accumulation of SSNA1 on MT ends correlates with suppression of MT growth rate, functioning as a potent MT-stabilizing protein (Lawrence et al., 2021). Curiously, SSNA1 also senses MT lattice damages induced by the severing enzyme spastin. Indeed, SSNA1 is a binding partner of spastin (Errico et al., 2004) with both proteins co-localizing and promoting axonal branching (Yu et al., 2008; Goyal et al., 2014; Basnet et al., 2018). SSNA1 binding to MTs protects them against spastin’s severing, similar to what happens with tau islands (Siahaan et al., 2019; Tan et al., 2019).

CRMPs (CRMP1-5) are a group of multifunctional proteins involved in neuronal development and regeneration (Nakamura et al., 2020). Recent studies revealed the interaction between different CRMPs and spastin in the modulation of MT dynamics, neurite outgrowth and axon regeneration (Ji et al., 2018; Ji et al., 2021; Li et al., 2021). CRMP2 interacts with the MTBD of spastin (Li et al., 2021). In hippocampal neurons, phosphorylation of spastin decreases its MT severing efficiency and thus weakens the cooperative effect of CRMP2 and spastin interaction with a negative effect in branch formation and neurite outgrowth (Li et al., 2021). Spastin also interacts with CRMP3 co-localizing with it *in vitro* and *in vivo*. Primary hippocampal neurons co-transfected with CRMP3 and spastin show enhanced neurite length and total number of branching neurites (Ji et al., 2021). Finally, spastin also binds CRMP5 through amino acid residues 270–328 (that correspond to the MTBD) (Ji et al., 2018). Spastin and CRMP5 cooperate to regulate MT dynamics and neurite outgrowth, as MTs are not severed by spastin when it is co-transfected with CRMP5.

In addition to MAPs, RhoA negatively modulates neurite outgrowth by regulating katanin and spastin (Tan et al., 2020). RhoA-ROCK is a well know inhibitory pathway of neurite outgrowth leading to growth cone collapse by stabilizing the actin cytoskeleton (Kalpachidou et al., 2019). In primary cultures of dorsal root ganglia neurons, inactivating RhoA, and its downstream effector ROCK, significantly increases the mRNA and protein levels of spastin and katanin p60. As such, RhoA not only regulates the actin cytoskeleton, but it also modulates MT dynamics by controlling the expression of severing enzymes, leading to the inhibition of axon (re)growth.

In summary, the levels and activity of spastin can be regulated by multiple mechanisms that together will contribute to the fine control of its function on the regulation of the MT cytoskeleton.

### Spastin Regulates Neuronal Biology Through Multiple Modes

Spastin is fundamental during neuronal development as it mediates axon branch loss in neuromuscular synapse elimination (Brill et al., 2016), and it sustains the transport of AMPA receptors and synaptic vesicles (Sherwood et al., 2004; Ji et al., 2020; Lopes et al., 2020; Li et al., 2021; Chen et al., 2022). Recently, spastin was shown to contribute to MT repair (Vemu et al., 2018; Kuo and Howard, 2021), extending the lifespan of axonal MTs by rejuvenating and protecting them against depolymerization. Spastin is also involved in the axonal transport of different organelles, with spastin knockout models displaying several transport impairments, such as axonal swellings and cargo stalling (Tarrade et al., 2006; Fassier et al., 2013; Denton et al., 2014). Its role in regulating axonal transport together with the modulation of ER and endosome fission and trafficking influence axonal branching (Yu et al., 2008) and axon (re)grow (Shirane and Nakayama, 2006; Rao et al., 2016; Petrova et al., 2020). In the following sections we discuss the different ways in which spastin participates in axon biology.

### Spastin Underlies Synapse Elimination, Synapse Density and Transport of Synaptic Cargoes

Throughout development, neurons form numerous synaptic connections that are then pruned during postnatal maturation (Lee, 2020). This process eliminates redundant synaptic connections, until only one single axon branch remains. Being essential for proper wiring and function of a mature nervous system, pruning failure is on the genesis of different neurodevelopment and neuropsychiatric disorders (Fu et al., 2012; Lai et al., 2012). In mouse neuromuscular junctions, Brill and colleagues (Brill et al., 2016) uncovered that spastin is involved in the biological events that precede the elimination of single axon branches during postnatal pruning. By tracing labeled mitochondria and peroxisomes, they observed absence of axonal transport in losing axon branches when compared with winning axon branches, which maintain bi-directional trafficking. Furthermore, losing axon branches displayed less tubulin and higher MT dynamics, suggesting MT cytoskeleton fragmentation and destabilization. Finally, they identified spastin as a central molecule underlying this process, as spastin knockout mice exhibited a reduction in the speed of shortening of losing axon branches, that presented reduced MT degradation. How spastin activity is tightly regulated in a branch-specific manner is still under investigation. This study pointed out to the role of tubulin polyglutamylation in this process. While wild-type animals exhibited less polyglutamylated tubulin in losing axon branches, suggesting an increased activity of spastin in these branches, spastin knockout mice had increased polyglutamylated tubulin probably due to the lack of spastin’s severing activity. Slightly different from mouse models, a previous study using spastin loss-of-function in *Drosophila* revealed the presence of more synaptic boutons and synaptic transmission impairment in spastin mutant larvae than in wild-type larvae (Sherwood et al., 2004). However, and in contrast to the spastin knockout mouse model discussed previously, spastin loss-of-function in *Drosophila* lead to a depletion in MT bundles at distal synapses, supporting a role of spastin in MT nucleation in this particular model.

**FIGURE 1:**
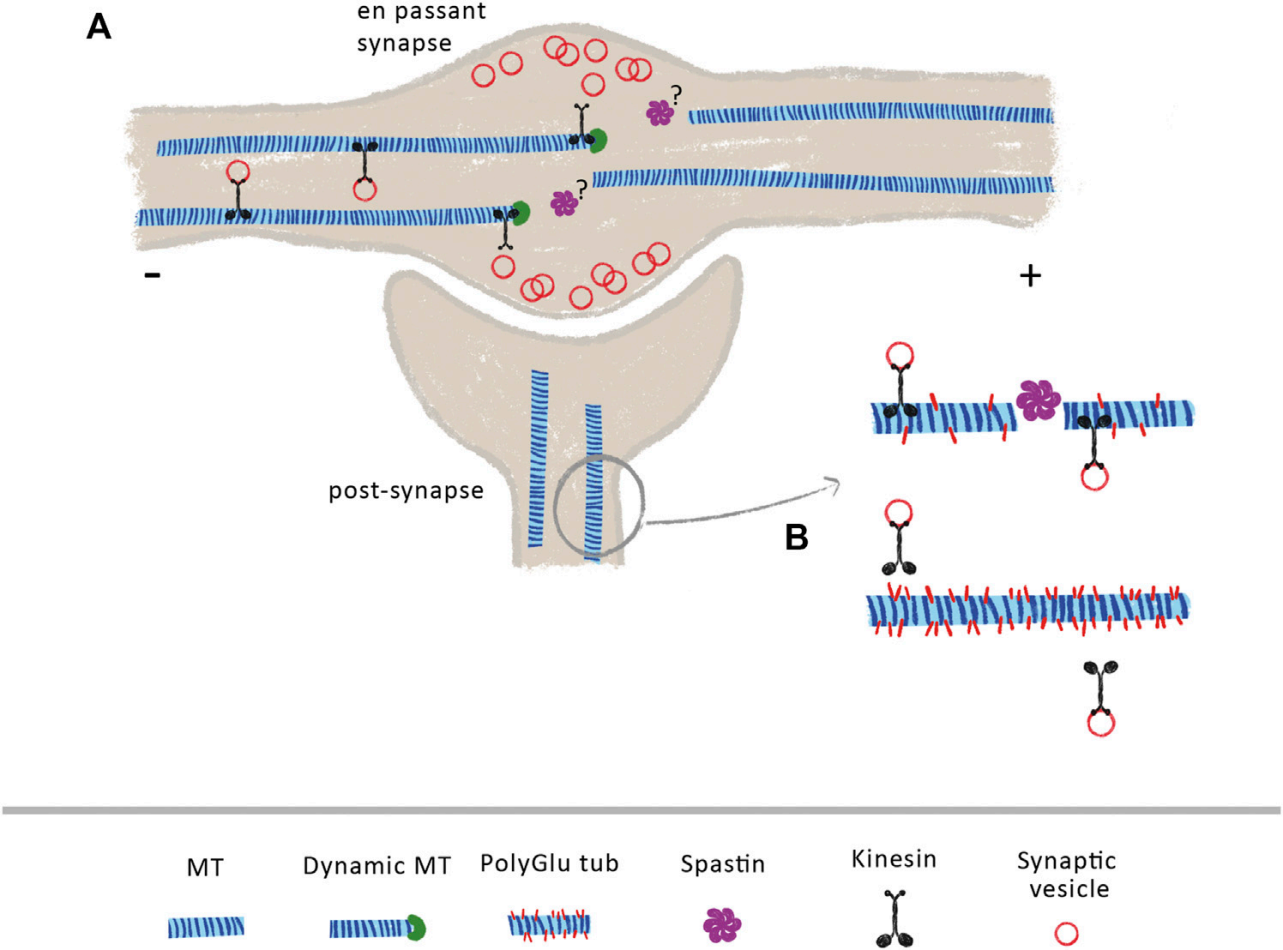
Spastin at pre- and post-synaptic sites **(A)** At axonal *en passant* synapses, the axonal transport of synaptic vesicles is dependent on the increase dynamics of MTs. Due to the weak affinity of kinesin-3 to MT GTP-bound tubulin, these molecular motors detach from MTs when they encounter their dynamic plus-ends, releasing transported cargoes within the synapse. The mechanisms underlying increase MT dynamics at these sites are currently under investigation. Due to its role in MT dynamics, the control of spastin activity at axon pre-synapses might contribute to generate a pool of dynamic MTs **(B)** At post-synapses, spastin indirectly modulates the transport of synaptic vesicles and AMPA receptors through the control of tubulin polyglutamylation levels. Loss of spastin increases tubulin polyglutamylation levels, decreasing the binding affinity of KIF5, leading to transport impairments.

Once established, presynaptic terminals and *en passant* synapses along axons require both the continuous delivery of synaptic components and the clearance of aged components. Both supply and clearance rely on a proper axonal transport and MT organization (Guedes-Dias and Holzbaur, 2019). Axonal MTs are densely packed along the axon shaft, becoming sparse at growth cones and axonal termini (Burnette et al., 2008; Nirschl et al., 2016; Yogev et al., 2016). In presynaptic areas, MTs are more dynamic and shorter to promote cargo pausing and delivery (Hendricks et al., 2010; Guedes-Dias et al., 2019; Qu et al., 2019). Indeed, in cultured mouse hippocampal neurons, *en passant* synapses depend on the weak affinity between kinesin-3 and synaptic MTs plus-ends to correctly deliver synaptic vesicles (Figure 1A) (Guedes-Dias et al., 2019). Since spastin regulates MT dynamics and nucleation (Vemu et al., 2018; Kuo et al., 2019), it is possible that it might play an important role in regulating MT dynamics at presynaptic sites in vertebrates (Figure 1A). Supporting this hypothesis, while wild-type Drosophila larvae display neuromuscular junctions with MTs arranged in loops and bundles, spastin-null larvae display diffuse patterns, denoting the fundamental role of spastin at axonal presynapses (Sherwood et al., 2004). Additionally, other studies show that the absence of spastin at axonal presynaptic sites causes excessive stabilization of MTs, causing defects in synapse growth and neurotransmission (Trotta et al., 2004; Orso et al., 2005).

Different reports support that spastin is also important in postsynaptic terminals. Loss of spastin in mouse hippocampal neurons leads to a reduction in synapse density and to increased levels of polyglutamylated tubulin (Lopes et al., 2020). Importantly, in these hyperglutamylation conditions, the binding affinity and processivity of the molecular motor KIF5 decreases, leading to deficits in the transport of synaptic vesicles and AMPA receptors (Figure 1B). Interestingly, reducing polyglutamylated tubulin on a spastin knockout background normalizes KIF5 transport and attenuates loss of synapses (Lopes et al., 2020). This work demonstrates that spastin is important not only to maintain synapse density, but also to indirectly regulate synaptic transport through the maintenance of low polyglutamylated tubulin levels. Synaptic plasticity is regulated by the traffic of AMPA receptors at postsynapses (Forrest et al., 2018). Recently, spastin was shown to directly interact with all the four subunits of AMPA receptors, in cultured rat hippocampal neurons, with phosphorylation of spastin at Ser210 enhancing its binding and surface delivery of AMPA receptors at synapses, in a mechanism independent of MT dynamics (Chen et al., 2022).

### Spastin Contributes to Axonal MT Longevity by Supporting Tubulin Self-Repair

As previously referred to, the severing enzymes katanin and spastin can introduce nanodamage on the MT lattice by actively extracting tubulin heterodimers out of the MT (Mcnally and Roll-Mecak, 2018; Vemu et al., 2018; Kuo and Howard, 2021). These nanodamages created along the MT can be repaired by spontaneous *de novo* incorporation of GTP-bound tubulin, forming GTP-islands (Vemu et al., 2018). If the local activity of severing enzymes is sufficiently low that it allows the passive incorporation of GTP-bound tubulin heterodimers, these new GTP-islands created along the MT lattice hold the possibility to rejuvenate MTs, stabilizing them against depolymerization. Additionally, incorporation of GTP-bound tubulin at severing sites ensures that, in case of a severing event, the newly formed MT emerges with a stable plus-end that protects it from spontaneous depolymerization, promoting MT elongation. This only remains true if GTP-bound tubulin does not readily hydrolyze into GDP-bound tubulin, which still needs to be experimentally confirmed. The possibility that spastin and katanin may play an important role in increasing the lifespan of MTs is particularly important in axonal MTs due to their extreme lengths and heavy dependence on long-range transport. MT repair is not randomly distributed along the MT length but preferentially located in regions where the lattice is likely submitted to geometrical and mechanical constrains, such as MT crossover, bundle or bending sites (Aumeier et al., 2016). Interestingly, these are also sites where severing enzymes are highly recruited (Lindeboom et al., 2013; Zhang et al., 2014). This suggests that sites of GTP-bound tubulin incorporation occur as a consequence of the action of MT-severing enzymes and correspond to genuine repair sites. Rejuvenating MTs with new GTP-islands through severing activity may additionally have a substantial impact in modulating transport led by different molecular motors, that may have differential binding to GTP-bound tubulin (Nakata et al., 2011).

### Spastin Regulates Axonal Transport by Interfering With MT Organization and Dynamics

Long-distance axonal transport is fundamentally dependent on the MT cytoskeleton. For this reason, the regulation MT organization and dynamics by MAPs and PTMs, is directly connected with the effectiveness of axonal transport. A dysfunctional MT cytoskeleton is associated with different neuronal diseases with impaired cargo transport (Roy et al., 2005; De Vos et al., 2008; Millecamps and Julien, 2013; Bodakuntla et al., 2021). One of the strongest pieces of evidence supporting the role of spastin in the modulation of axonal transport came from observations in HSP-SPG4 patients, as one of the main pathological cellular hallmarks is axonal transport impairment (Tarrade et al., 2006). In this respect, HSP-SPG4 axonal swellings show a disorganized MT cytoskeleton with accumulation of organelles, a sign of impaired MT dynamics and axonal transport. In induced pluripotent stem cells derived from HSP-SPG4 patients, axonal swellings exhibit accumulation of mitochondria and tau (Denton et al., 2014). Mouse models carrying spastin mutations also show focal axonal swellings, in both ascending and descending tracts (Tarrade et al., 2006; Kasher et al., 2009). These swellings have a disorganized MT cytoskeleton with impaired dynamics, which alters axonal transport increasing the frequency of cargo stalling (Fassier et al., 2013). In fact, spastin’s severing activity indirectly regulates the transport of mitochondria (Mcdermott et al., 2003; Tarrade et al., 2006; Kasher et al., 2009), peroxisomes (Tarrade et al., 2006; Wali et al., 2016; Wali et al., 2020), amyloid precursor protein-vesicles (Kasher et al., 2009), lysosomes (Fuerst et al., 2011), vesicle-associated membrane protein 7 (Plaud et al., 2017), and ER (Rao et al., 2016). Although both anterograde and retrograde transport present defects, cargo velocities vary considerably between spastin mutations (Fuerst et al., 2011). The importance of the regulation of MT dynamics by spastin and the efficiency of axonal transport, was reinforced by the fact that tubulin-binding drugs that interfere with MT stability in conditions in which spastin is depleted, restore axonal transport impairments (Fassier et al., 2013; Denton et al., 2014; Fan et al., 2014; Wali et al., 2016; Wali et al., 2020). Furthermore, in the absence of spastin, increased levels of polyglutamylated tubulin are found in mouse cultured hippocampal neurons (Lopes et al., 2020), which leads to impairments in the transport of synaptic cargoes. This study underlies that spastin indirectly regulates the transport of certain cargoes through the modulation of tubulin PTM levels.

**FIGURE 2:**
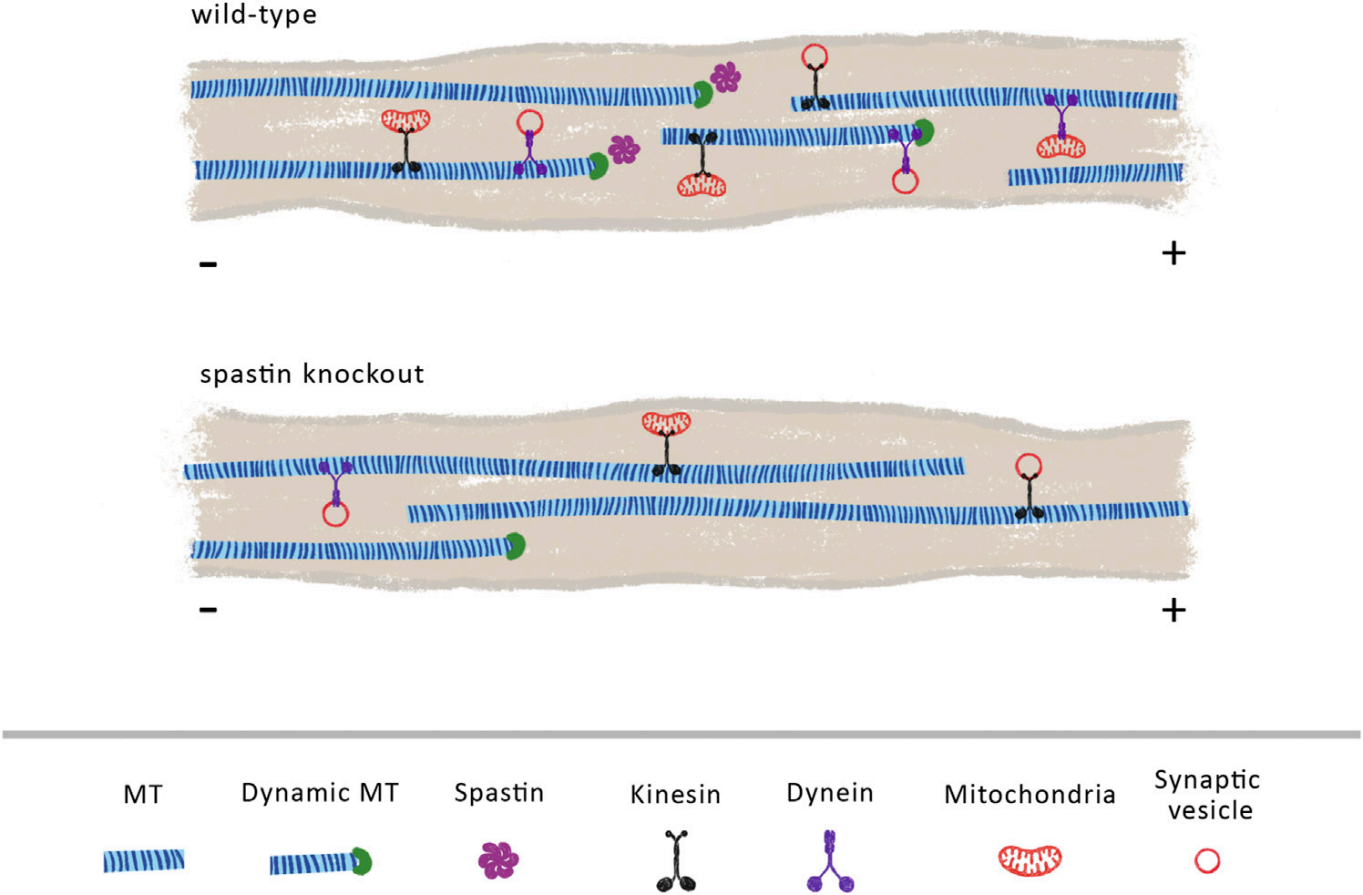
Spastin plays a vital role in regulating MT cytoskeleton density and dynamics. We propose that in wild-type animals, endogenous spastin contributes to the maintenance of a dynamic and dense MT cytoskeleton that sustains an efficient axonal transport. Its activity may increase the number of dynamic MTs, facilitating the loading of retrograde cargoes mediated through dynein motors. In the absence of spastin, the density and dynamics of MTs is reduced, due to a decline in the levels of MT severing and MT nucleation, decreasing the overall axonal transport.

Axonal transport is heavily dependent on MT organization and dynamics. Through its severing and MT nucleation activity (Vemu et al., 2018; Kuo et al., 2019) it is possible that spastin increases the dynamicity and density of MTs (Figure 2). As supported by previous reports, increasing MT density enhances axonal transport due to a reduction in the time that cargoes spend switching between MT polymers (Yogev et al., 2016). Increasing MT density also decreases the space between parallel MTs, enhancing motor engagement with more than one MT polymer, which, consequently, boosts motor activity and axonal transport (Wortman et al., 2014). Moreover, by increasing MT dynamics, spastin might indirectly regulate retrograde axonal transport of dynein-mediated cargoes (Figure 2). To initiate its transport on MTs, dynein binds to dynactin, a multi-subunit complex required to activate dynein-driven transport along the axon (WatermanStorer et al., 1997). The initiation of dynein-driven transport is dependent on the affinity of dynactin for tyrosinated MTs, being recruited to the dynamic plus-end of MTs (Nirschl et al., 2016). Therefore, a possible role of spastin in the maintenance of a pool of dynamic MTs at distal regions of axons is critical for dynein-based transport (Moughamian and Holzbaur, 2012). Supporting this hypothesis, different animal models show that the absence of spastin results in decreased MT density and dynamics (Sherwood et al., 2004; Trotta et al., 2004; Wood et al., 2006). Future experiments need to further investigate the role of spastin in regulating distal retrograde axonal transport and increased axonal transport through the control of MT density.

### Spastin Regulates Axon Branching and (re)Growth Though Mechanisms Dependent and Independent of MT Modulation

**FIGURE 3:**
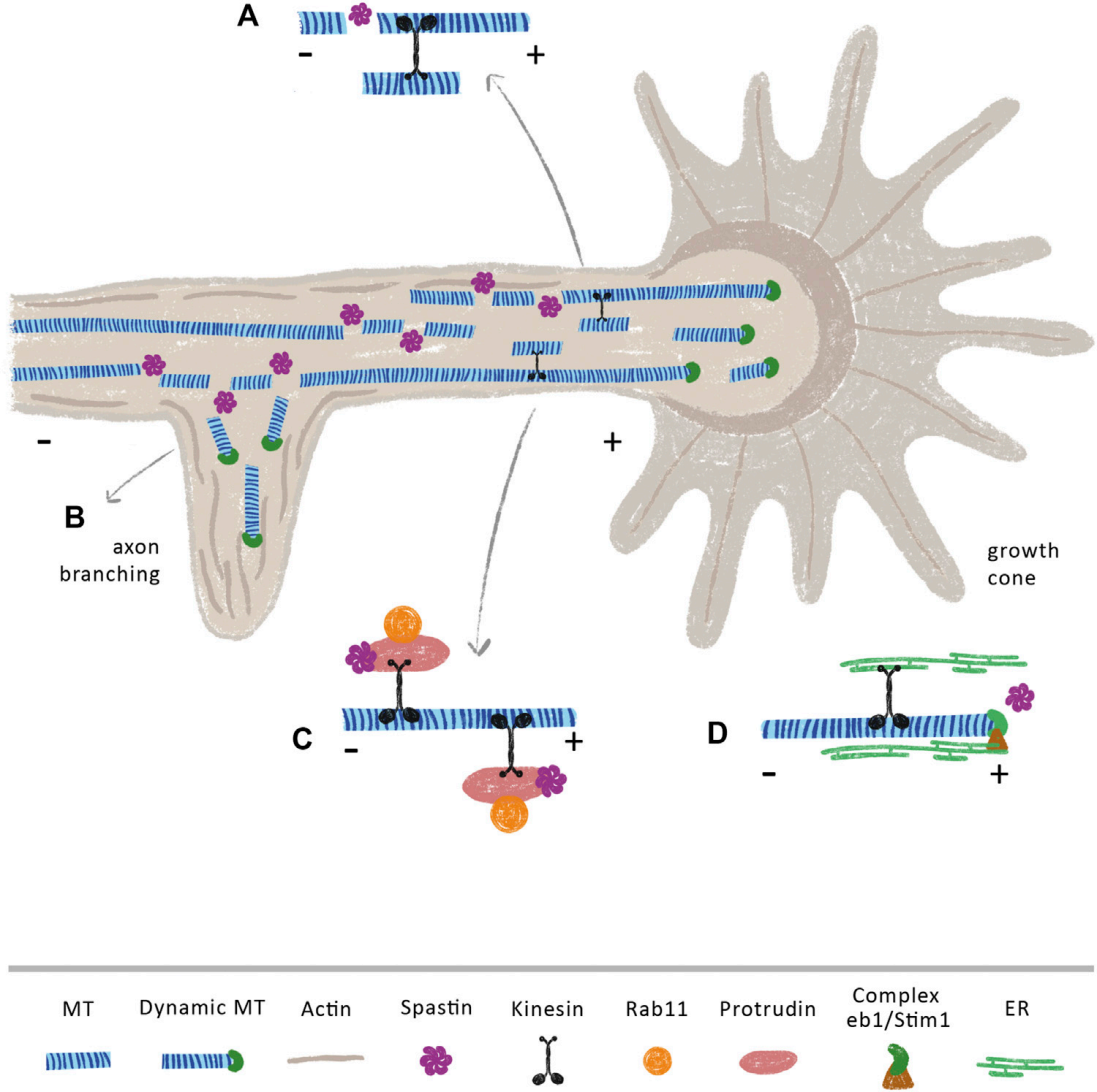
Spastin modulates axon branching and (re)growth through MT dependent and independent mechanisms. Spastin severing activity may increase the availability of short and dynamic MTs that are anterogradely transported towards **(A)** growth cones and **(B)** axonal branches, supporting axon elongation at these sites. Of note, spastin is highly concentrated at these sites (Yu et al., 2008) **(C)** Spastin regulates the transport of the growth-related molecule Rab11 by interacting with the ER protein protrudin. In CNS, overexpression of protrudin leads to robust CNS regeneration (Petrova et al., 2020) **(D)** The movement of ER through the tip attachment complex is mediated by the binding of ER to the protein complex STIM1 and EB1 at the plus-tip of MTs. Upon injury, the ER accumulates at growth cones of regenerating axons (Rao et al., 2016). We propose that an increase of spastin after injury, may increase the number of MT plus-tips, which might underlie the increment in the transport of ER to these axonal sites.

Axon branching and outgrowth are vital to ensure proper brain connectivity and development. While axon outgrowth is related with axon guidance and pathfinding, branching is crucial to create intertwining neuronal circuits (Mccormick and Gupton, 2020; Hoersting and Schmucker, 2021). The process of axon outgrowth and axon branching share some similarities. At the tip of the axon, the growth cone powers axon elongation (Figure 3). A tight regulation of the MT cytoskeleton is essential to sustain an efficient growth cone, which needs to be at the same time dynamic in its peripheral domain, in order to explore the extracellular environment, and stable in its central domain to be capable to propel axon elongation (Song and Brady, 2015). On the other hand, during the formation of axon branches, after the extension of plasma membrane protrusions filled with actin patches (Spillane et al., 2011), axonal MTs move in to stabilize the newly formed branches (Dent and Kalil, 2001) (Figure 3B). In fact, short MTs are severed from bundles of MTs in growth cones (Dent et al., 1999), creating a pool of local dynamic MTs that will then be used to produce MT advancements during axon elongation (Baas et al., 2005; Baas et al., 2006) (Figure 3A). Similarly, during branch formation, early studies demonstrate that MTs in the axon shaft undergo destabilization with fragmented MTs being transported to or generated at newly forming branches, enabling their growth (Figure 3B) (Yu et al., 1994; Gallo and Letourneau, 1999; Armijo-Weingart et al., 2019). Due to their cutting capacity, severing enzymes are strong candidates to support the formation of these short and mobile MTs. Supporting this idea, spastin was shown to be highly concentrated at growth cones and sites of branch formation in cultured rat hippocampal neurons, with spastin overexpression dramatically enhancing branch formation due to generation of short MTs (Yu et al., 2008). Similarly, in zebrafish, knockdown of spastin severely impairs early neuronal development by weakening the formation of MT networks that are essential for axon growth and arborization (Wood et al., 2006). Moreover, cultured hippocampal neurons derived from human induced pluripotent stem cells of patients carrying SPAST mutations, exhibit reduced neurite length and branching when compared with control neurons derived from healthy patients (Havlicek et al., 2014). This study also observed an increase in katanin p60 mRNA and protein expression levels, indicating that SPG4-derived neurons partially compensate for the loss of spastin by upregulating katanin, which, to some extent, may support the regulation of MT dynamics. However, this increase in katanin is not sufficient to rescue the abnormal neurite phenotype pointing out that spastin may have specific functions that cannot be replaced by other severing enzymes. Of note, overexpression of spastin can produce different effects depending on gene dosage. While overexpressing human spastin under the strong CMV promoter causes a time-dependent neurite shortening and branching in cultured mouse hippocampal neurons, using the endogenous spastin promotor elicits the formation of a higher number of long and complex neurites (Riano et al., 2009). Interestingly, SSNA1 accumulates at axon branching sites, protecting MTs from the action of spastin, with its overexpression inducing branch formation (Basnet et al., 2018). This suggests that SSNA1 might be a specific regulator of spastin at branch formation sites, regulating the tight balance between MT dynamics and stabilization through modulation of this severing enzyme.

Multiple studies support that beyond axon growth and branching, spastin is also important during axon regeneration. While in *Drosophila*, spastin is necessary for axon regeneration but not for dendrite regrowth (Stone et al., 2012), in rats, delivery of spastin protein immediately after sciatic nerve transection enhances sensory fiber regeneration and motor function recover (Lin et al., 2017). At the molecular level, it has been demonstrated that events essential for neurite outgrowth and regeneration are mediated by the interaction of spastin with several ER proteins, in MT-dependent and independent manners. Axons are composed of smooth ER, a network of interconnected cylindrical tubules with occasional small cisternae. ER tubules dynamically change their morphology and position within the cells in response to physiological stimuli, being these arrangements mediated by the MT cytoskeleton. Protrudin is an integral ER membrane protein that functions as a scaffold with interaction sites for key axon growth-related molecules, such as Rab11 and kinesin-1 (Shirane and Nakayama, 2006). In the context of axon regeneration, the spastin-protrudin interaction enables an increase in the anterograde transport of Rab11-positive recycling endosomes, facilitating axon repair (Figure 3C) (Shirane and Nakayama, 2006; Zhang et al., 2012). Reducing the levels of either protrudin or spastin severely impairs the outgrowth of zebrafish spinal or branchiomotor neuronal axons (Zhang et al., 2012). Of note, protrudin is expressed at low levels in CNS neurons, but at higher levels in regenerating PNS neurons; its overexpression leads to robust CNS regeneration *in vitro* and *in vivo* through increased transport of Rab11 endosomes, integrins, and accumulation of ER at the axon tip (Petrova et al., 2020). Spastin physically interacts with other ER proteins, for instance, atlastin and REEP1 (Evans et al., 2006; Sanderson et al., 2006; Park et al., 2010). Spastin, atlastin and REEP1 interact with each other via hydrophobic hairpins within the tubular ER to coordinate its shape and its interaction with MTs. Interestingly, mutations in genes that encode atlastin and REEP1 also lead to HSP (Lo Giudice et al., 2014), suggesting that these three proteins—atlastin, REEP1 and spastin—might work together to form the ER network. Along these lines, in *Drosophila*, spastin cooperates with atlastin, increasing the concentration of ER, together with underlying MTs, at tips of regenerating axons, but not at the tips of regenerating dendrites, supporting axon regeneration (Rao et al., 2016). In both vertebrates and invertebrates, ER tends to co-align with MTs, which drives the movement of ER tubules (Terasaki et al., 1986; Kimura et al., 2017; Farias et al., 2019). The movement of ER can occur either by a tip attachment complex (TAC) or by ER sliding. In TACs, the tip of an ER tubule is attached to the tip of a MT plus-end through the ER protein STIM1 and the MT-binding protein EB1 (Grigoriev et al., 2008; Pavez et al., 2019) (Figure 3D). By increasing MT dynamics, spastin may increase the density of MT plus-ends affecting the transport of these structures along the axon. Since an increase in ER at the growth cone is necessary to support axon regeneration (Rao et al., 2016), it is possible that local spastin regulation upon injury might be fundamental to increase ER transport by increasing the availability of MT plus-ends. Given their different regenerative capacities (Qian and Zhou, 2020), it would be important to investigate if a distinct regulation of spastin occurs in central and peripheral neurons.

Spastin mutations that hyper-stabilize MTs also disturb the balance between ER tubules and cisternae in *Drosophila* neurons (Vajente et al., 2019). This hyper-stabilization results in reduced ER calcium content, affecting the flight ability of flies. Importantly, restoring MT dynamics by administering the MT-destabilizing drug vinblastine, rescues ER morphology, calcium storage and flight ability (Vajente et al., 2019). This study demonstrates that spastin affects the MTs surrounding the ER, impacting its morphology and function in neurons. Calcium release from the ER upon an injury is essential to the transcription of regenerative-associated genes (Cho et al., 2013), membrane resealing (Tuck and Cavalli, 2010), protein synthesis (Perry and Fainzilber, 2014), and MT reorganization upon a nerve injury (Ghosh-Roy et al., 2012; Valakh et al., 2013). If spastin mutations reduce ER calcium storage and impair store-operated calcium, they will plausibly affect the regenerative capacity of neurons. Importantly, many of these events are impaired in central axon regeneration, which raises the question if lower spastin activity underlies, at least in part, the lack of regenerative capacity seen in these neurons.

The axonal ER also has several membrane contact sites with the plasma membrane and other membrane-bound organelles, such as mitochondria, vesicles and endosomes, which can indirectly influence the regulation and transport these organelles along MTs (Wu et al., 2017). The role of endosomes in axon regeneration has been widely studied, and Rab11, a protein that controls endocytic vesicular trafficking, is known to target the transport of several growth-related molecules (Shirane and Nakayama, 2006; Petrova et al., 2020; Zhang et al., 2021). Until recently, the mechanisms involved in the fission of endosomes from ER membrane contact sites and the consequences of its failure were unknown. Different research studies found that both spastin isoforms M1 and M87 appear to act in concert to drive efficient fission of endosome tubules from early sorting endosomes (Connell et al., 2009; Allison et al., 2013; Allison et al., 2017). This process is mediated by spastin MIT domain (Reid et al., 2005; Allison et al., 2017). The absence of spastin has a direct impact in the efficient sorting of membrane receptors, with cortical neurons from spastin-HSP mice and iPSC-derived patient neurons exhibiting failure in trafficking receptors via the tubular-vesicular pathway (Allison et al., 2013; Allison et al., 2017). The missorting of these receptors leads to their transfer to endolysosomes, causing defective lysosomal enzyme traffic accompanied by abnormal lysosomal morphology. Actually, in the absence of spastin, lysosomes increase in size, become more acidic, accumulate dense membranous material and reduce in number (Allison et al., 2017; Newton et al., 2018). Spastin can also regulate endosome trafficking by antagonizing protrudin (Connell et al., 2020). ER localized protrudin interacts with Rab7 and phosphatidylinositol 3-phosphate. These contacts facilitate transfer of KIF5 that is also captured by protrudin, to the late endosomal motor adaptor FYCO1, promoting MT-motor-dependent movement of endosomes towards the plasma membrane.

Collectively, the above studies show that MT severing enzymes are fundamental regulators of axon growth, branching and regeneration, through mechanisms that are dependent or independent of MT regulation.

### A Final Note on Hereditary Spastic Paraplegia

HSP is a group of heterogeneous neurodegenerative disorders characterized by the progressive length-dependent distal axonopathy of descending motor nerve fibers of corticospinal tracts (upper motor neurons) and ascending dorsal columns (sensory neurons) within the spinal cord, resulting in slow and progressive weakness of lower extremities and spasticity (Elsayed et al., 2021; Chaudhary et al., 2022). HSPs are the second most frequent motor neuron disease with an estimated prevalence of 3–10/100,000 (Noreau et al., 2014). More than 100 loci and 88 genes are implicated in the pathogenesis of HSP, being SPAST, the gene that encodes for the protein spastin, the most frequently mutated HSP gene (termed HSP-SPG4), accounting for more than 50% of autosomal dominant familial and 20% of sporadic HSP cases (Chaudhary et al., 2022). Future research should also expand the causes of HSP towards noncoding genomic sequences and environmental factors in order to improve genetic diagnoses and therapies (Bis-Brewer and Zuchner, 2018). In HSP-SPG4, spastin is mutated in more than 200 sites distributed along the gene sequence (except for the exon 4, which undergoes alternative splicing), including point mutations, frameshift mutations, large deletions, and missense mutations (Depienne et al., 2007; Shoukier et al., 2009). Given that the same disease arises from different types of mutations and that no mutant spastin is detected in cells and tissues from human patients outside the CNS (Svenson et al., 2001; Abrahamsen et al., 2013; Solowska and Baas, 2015), a loss-of-function (i.e., haploinsufficiency) etiology for the disease was promptly suggested. However, and contrary to humans, knockout mouse models develop mild and late onsets in motor defects, without showing neuron degeneration. Homozygous spastin knockout mice present axonal swellings, axonal transport impairments and reduced MT dynamics, not recapitulating all the features of the human disease, which include corticospinal degeneration and progressive axon die-back (Tarrade et al., 2006; Kasher et al., 2009). Additionally, since HSP-SPG4 does not affect the fetus and almost exclusively impacts motor neurons, the haploinsufficiency theory fails to explain why this happens, given that if the disease was only explained by a loss-of-function of spastin, defects should probably appear during embryogenesis as well as across other neuronal populations.

In contrast to models where spastin is absent, a mouse model expressing human mutant spastin exhibits severe adult-onset gait deficiency and corticospinal dieback degeneration, while maintaining normal levels of endogenous spastin (Qiang et al., 2019b). Nevertheless, this model also shows the lack of some human HSP-SPG4 characteristics, such as the development of axonal swellings. These contradictory results raise the hypothesis that HSP-SPG4 evolve not only through loss-of-function or gain-of-function mechanisms, but through both (Mohan et al., 2021). In fact, experimental data reveals that a gain-of-function mechanism is exacerbated by the spastin haploinsufficiency (Qiang et al., 2019b). This study shows that cultured neurons from mice with carrying a human spastin mutation display deficits in axonal transport which are worsened by the depletion of endogenous spastin. Indeed, mutations in spastin M1 were shown to be neurotoxic, with mutant spastin heavily decorating MTs, decreasing its dynamics, inhibiting fast axonal transport, and impairing axonal growth (Solowska et al., 2008; Solowska et al., 2014; Leo et al., 2017). Additionally, mutant forms of spastin M1 abnormally and specifically activate casein kinase 2, affecting axonal transport by causing organelle distribution defects (Leo et al., 2017). As seen before, while M87 is widely distributed spastin M1 is only detectable in the adult CNS (Solowska et al., 2008). Since the mutant spastin M1 tends to be more stable than the mutant spastin M87, this raises the assumption that mutated spastin M1 specifically accumulates in the CNS motor neurons, where it is heavily expressed (Solowska et al., 2017). This might explain, at least in part, why motor neurons are more prone to be affected when mutations in this protein occur. Their long lengths and complex synapse arborization also pose a high dependence on an efficient axonal transport, MT maintenance, receptor recycling, and protein and lipid synthesis. As explored in this review, spastin participates in all these cellular functions which may render this neuron type particularly susceptible to spastin mutations. Finally, the gain-of-function hypothesis may also underlie the fact that HSP does not cause fetal abnormalities, as the spastin M1 isoform is only highly expressed during adult CNS development.

## Conclusion

Synapse formation and maintenance, MT repair, axonal transport, branching and (re)growth are all processes dependent on the tight balance of MT dynamics. In this review we provide an integrative view on recent studies developed in multiple model organisms supporting that MT severing enzymes in general, and spastin in particular, are important players underlying optimal axon cell biology. Future research should further dissect the molecular mechanisms of spastin action, its possible local regulation, and binding partners, to further unveil the role of this MAP as a facilitator of axon (re)growth.
